# Pharmacogenomics of antiepileptic drug mood stabilizer treatment response in bipolar disorder: A MoStGen Consortium study

**DOI:** 10.1038/s41380-026-03478-7

**Published:** 2026-02-19

**Authors:** Ada Man-Choi Ho, Brandon J. Coombes, Anthony Batzler, Vanessa K. Pazdernik, Richard S. Pendegraft, Michelle Skime, Narjes Bendjemaa, Bernardo Carpiniello, Martina Contu, Nina Dalkner, Frederike T. Fellendorf, Giovanna Fico, Claudio D. Fullerton, Manuel Gardea-Resendez, Sarai Gonzalez-Garza, Nematollah Jaafari, Esther Jiménez, Oussama Kebir, Adrien Legrand, Sofia Luna-Garza, Anna Meloni, Bruno Millet, Fayçal Mouaffak, Abraham Nunes, Claire O’Donovan, Pasquale Paribello, Marco Pinna, Claudia Pisanu, Edith Pomarol-Clotet, Francisco Romo-Nava, Raúl F. Sánchez, Katie Scott, Alessio Squassina, Elisabet Vilella, Alessandro Serretti, Miguel L. Prieto, Eva Z. Reininghaus, Susanne A. Bengesser, Alfredo B. Cuellar-Barboza, Marie-Odile Krebs, Boris Chaumette, Eduard Vieta, Mirko Manchia, Susan L. McElroy, Martin Alda, Mark A. Frye, Joanna M. Biernacka

**Affiliations:** 1 Department of Psychiatry and Psychology, Mayo Clinic, Rochester, MN USA; 2 Department of Quantitative Health Sciences, Mayo Clinic, Rochester, MN USA; 3 GHU Paris Psychiatrie et Neurosciences, Hôpital Sainte Anne, Paris, France; 4 Section of Psychiatry, Department of Medical Sciences and Public Health, University of Cagliari, Cagliari, Italy; 5 Unit of Clinical Psychiatry, University Hospital Agency of Cagliari, Cagliari, Italy; 6 Division of Psychiatry and Psychotherapeutic Medicine, Medical University Graz, Graz, Austria; 7 Institute of Neuroscience, University of Barcelona, Barcelona, Catalonia Spain; 8 Hospital Clinic, IDIBAPS, CIBERSAM, Barcelona, Catalonia Spain; 9 Department of Psychiatry, Faculty of Medicine, Universidad de los Andes, Santiago, Chile; 10 Mental Health Service, Clínica Universidad de los Andes, Santiago, Chile; 11 Department of Psychiatry, School of Medicine, Universidad Autonoma de Nuevo Leon, Monterrey, Mexico; 12 Unité de Recherche Clinique Intersectorielle en Psychiatrie, Centre Hospitalier Henri Laborit, Poitiers, France; 13 Centro de Investigación Biomédica en Red de Salud Mental, CIBERSAM-ISCIII, Madrid, Spain; 14 Université Paris Cité, Institute of Psychiatry and Neuroscience of Paris (IPNP), INSERM U1266, Paris, France; 15 Section of Neuroscience and Clinical Pharmacology, Department of Biomedical Sciences, University of Cagliari, Cagliari, Italy; 16 Infrastructure of Clinical Research in Neurosciences-Psychiatry, Brain Institute (ICM), Sorbonne Université, INSERM, Paris, France; 17 Service de Psychiatrie Adulte de la Pitié-Salpêtrière, Sorbonne Université, Paris, France; 18 EPS Ville Evrard, Saint Denis, France; 19 Department of Psychiatry, Dalhousie University, Halifax, Nova Scotia Canada; 20 FIDMAG Germanes Hospitalàries Research Foundation, Barcelona, Spain; 21 Centro de Investigacion Biomedica en Red de Salud Mental, CIBERSAM-ISCIII, Barcelona, Spain; 22 Lindner Center of HOPE, Mason, OH USA; 23 Department of Psychiatry and Behavioral Neuroscience, University of Cincinnati College of Medicine, Cincinnati, OH USA; 24 Mood and Anxiety Clinical Center, Santiago, Chile; 25 Institut d’Investigació Sanitària Pere Virgili-CERCA, Reus, Spain; 26 Universitat Rovira i Virgili, Tarragona, Spain; 27 Hospital Universitari Institut Pere Mata, Reus, Spain; 28 Department of Medicine and Surgery, Kore University of Enna, Enna, Italy; 29 Oasi Research Institute-IRCCS, Troina, Italy; 30 Department of Psychiatry, McGill University, Montreal, Canada; 31 Department of Pharmacology, Dalhousie University, Halifax, Nova Scotia Canada; 32 National Institute of Mental Health, Klecany, Czech Republic

**Keywords:** Predictive markers, Genetics

## Abstract

Identifying biological and clinical factors associated with response to mood-stabilizing medications is critical for improving bipolar disorder (BD) treatment. The Mood Stabilizer Genomics (MoStGen) Consortium was established to investigate pharmacogenomic and clinical predictors of response to treatment of BD with antiepileptic drug mood stabilizers (AMS). Here we present the first pharmacogenomic analyses of AMS treatment outcomes based on MoStGen Consortium data, including 917 individuals across contributing sites. We performed genome-wide association analyses in subcohorts followed by meta-analyses, with AMS treatment response measured quantitatively using the Alda scale. Medication-stratified analyses were performed for valproic acid (VPA) and lamotrigine (LTG) treatment response. Additionally, polygenic score (PGS) analyses were used to evaluate the overall genetic contribution to AMS response across cohorts and to test whether genetic liability for various neuropsychiatric illnesses impacts AMS response. We detected genome-wide significant associations with LTG treatment response for SNPs in the gene *ROBO2* (top SNP: rs985123, *p* = 1.9E-10) and for *POLR1E* at the gene-level (*p* = 2.53E-06). No significant associations were found for overall AMS or VPA treatment response. Leave-one-out PGS analyses provided significant evidence for a polygenic signal for AMS treatment response. Furthermore, the epilepsy PGS was nominally significantly associated with AMS response (*p* = 0.024), suggesting higher genetic liability to epilepsy predicts a better response to treatment with AMS. These findings provide insights into the genetic contribution to AMS treatment outcomes, and in particular LTG response, and may contribute to the development of more precise treatments for BD.

## Introduction

Bipolar disorder (BD) is a severe mental illness characterized by recurrent episodes of mania/hypomania and depression with euthymic intervals, that causes a significant burden to patients and healthcare systems. It is highly complex and heterogeneous in clinical presentation, course of illness, prognosis, and medical comorbidities [1]. Responsiveness to mood-stabilizing pharmacotherapy, such as lithium, antiepileptic drug mood stabilizers (AMSs), and atypical antipsychotics, also varies greatly among patients with BD [2]. Therefore, identifying clinical characteristics and biomarkers that predict response (or non-response) to specific medications is an important step towards providing more effective and safe treatment for BD.

Several studies have aimed to identify pharmacogenomic markers for response to lithium in patients with BD [2–8]. The largest genome-wide association study (GWAS) of lithium response was conducted by the International Consortium on Lithium Genetics (ConLiGen) and considered lithium response as a quantitative trait measured using a validated scale developed by Martin Alda [9, 10]. The study included data from over 2500 European-ancestry patients with BD and found genome-wide significant associations between lithium response and genetic variants mapped to two long non-coding RNA genes [9]. Concerning lithium’s safety profile, a recent study of clinical and genetic predictors influencing its serum levels reported a genetic locus associated with lithium clearance adjusted for age and sex, but the addition of genetic markers did not significantly improve predictive models based on clinical predictors [11]. For AMSs, most prior pharmacogenomic studies focused on candidate genes and the medications’ pharmacokinetics while some investigated their side effects but rarely their treatment response in BD [12]. One GWAS of AMS treatment response has been published [13]. While the study included only 199 BD patients, single nucleotide polymorphisms (SNPs) in *THSD7A* and *SLC35F3* genes were found to be associated with AMS treatment response measured by the Alda scale. Furthermore, the study provided evidence of association with variants in *ABCC1* and *DISP1* at the gene level [13]. The Mood Stabilizer Pharmacogenomics (MoStGen) Consortium was subsequently established to facilitate pharmacogenomic and clinical analyses of AMS response, including treatment responses to specific AMSs (i.e. valproate and lamotrigine) in a large sample combining multiple international cohorts of patients with BD.

Here we report the first results of a GWAS of AMS (valproate/divalproex, lamotrigine, carbamazepine, and oxcarbazepine) treatment response in BD based on the current MoStGen Consortium dataset. Medication-stratified GWAS focusing on the two most commonly prescribed AMSs, valproate/divalproex and lamotrigine, are also presented. Finally, polygenic score (PGS) analyses are used to assess a polygenic contribution to AMS response in BD and to test whether genetic liabilities for various neuropsychiatric illnesses predict treatment response.

## Subjects and methods

### Study cohorts

The MoStGen Consortium contributing cohorts are described in the Supplementary Materials and include cohorts from North and South America and Europe. The consortium accrued data from 917 individuals with a diagnosis of bipolar spectrum disorder according to the Diagnostic and Statistical Manual of Mental Disorders (DSM) III, DSM-IV or DSM-5, basic demographic and clinical information, treatment response to AMS (valproate [VPA], lamotrigine [LTG], oxcarbazepine, or carbamazepine) measured using the Alda scale [9, 14], and genomic data. Participant characteristics are summarized in Table 1.

**Table 1:** Study participant characteristics.

Total *n*		917
Sex, *n* (%)		
Male		336 (36.64%)
Female		581 (63.36%)
Age at interview, mean (SD)		43.2 (14.1)
Genetic ancestry, *n* (%)		
European		809 (88.22%)
Latin American		70 (7.63%)
African American		25 (2.73%)
South Asian		7 (0.76%)
East Asian		6 (0.65%)
Bipolar type, *n* (%)		
Bipolar I		612 (66.74%)
Bipolar II		292 (31.84%)
Bipolar NOS		7 (0.76%)
Schizoaffective		6 (0.65%)
Alda scale: A score, mean (SD)		
Combined AMS (*n* = 895)		5.4 (2.7)
VPA (*n* = 546)		5.2 (2.9)
LTG (*n* = 446)		5.3 (2.6)
Alda scale: B score sum, mean (SD)		
Combined AMS (*n* = 895)		2.8 (1.3)
VPA (*n* = 528)		2.9 (1.4)
LTG (*n* = 436)		2.8 (1.3)
Alda scale: A score excluding B score sum > 4 (A_ex_ score)^†^, mean (SD)		
Overall (*n* = 810)		5.5 (2.6)
VPA (*n* = 472)		5.4 (2.8)
LTG (*n* = 399)		5.4 (2.6)

†Treatment outcome measure used in GWAS.

All contributors declared that all procedures involved in collecting and sharing the deidentified patient information submitted to the MoStGen Consortium complied with the Declaration of Helsinki and had been approved by their corresponding institutional ethics committees on human studies, and written consent had been obtained from all participants.

### Assessment of clinical response to AMS treatment

AMS treatment response was measured using the Alda scale, which has been validated and used by ConLiGen to retrospectively evaluate lithium treatment response in BD patients [9, 10, 14]. Briefly, the A score of the scale is a composite measure of improvement in frequency, duration, and severity of illness episodes ranging from 0 (no change or worsen) to 10 (complete response). The B score of the scale is composed of five items that ordinally quantify (from 0 to 2) the number of mood episodes before treatment (B1), the frequency of episodes before treatment (B2), duration of treatment (B3), medication compliance (B4), and the use of additional medication during periods of stability (B5). The B score sum provides an indicator of the degree of confidence in the A score (i.e., treatment response) since illness severity, treatment duration, medication compliance, and the influence of other psychotropic medications could confound the perceived treatment response. Higher B scores denote fewer and/or less frequent episodes before treatment, shorter treatment duration, poorer compliance, and/or a long-term/systemic use of antidepressants or antipsychotics. In this study, we used the A score after excluding individuals with a B score sum larger than 4 as the treatment response variable (A_ex_), which had been reported as the most reliable continuous phenotype derived from the Alda scale [9]. For individuals with outcome data for more than one AMS, the A score corresponding to the treatment episode with the lowest B score sum (and thus higher confidence in the A score) was used. If two or more medications had the same lowest B score sum, the A scores were averaged.

### Genotyping, quality control, and imputation

Genotyping was previously conducted at different sites using various assays (Table S1). Each cohort’s genotype data was processed using the Mayo Clinic genotype quality control pipeline. Individuals were removed if there were sex discrepancies, high genotype missingness ( > 5%) or low heterozygosity ( < 70%) on multiple chromosomes. Variants with high missingness ( > 5%), low frequency (MAF < 0.5%), or not in Hardy-Weinberg equilibrium (*p* < 1E-06) were removed. Genetic imputation was performed on each genotype dataset using the TOPMed Imputation Server [15] with the TOPMed reference panel. All data were then merged into one dataset retaining only variants with imputation dosage-R^2^ > 0.3 across all cohorts, and KING software [16] was used to assess relatedness among individuals across cohorts. One individual was removed from pairs with second degree or higher relatedness, retaining the individual with the lower Alda B score. ADMIXTURE software [17] was then used to estimate ancestry of the remaining individuals using the 1000 Genomes as a reference dataset [18], and genomic principal components (PCs) were computed using FlashPCA [19].

### GWAS and meta-analysis

GWAS analyses of treatment response to AMS were performed in five subsets of the data: three genotyped batches from the Mayo Clinic Bipolar Disorder Biobank (MCBDB), Halifax (four genotype datasets combined), and a dataset grouping cohorts with sample sizes too small to be analyzed separately denoted as CBPG (Cagliari, Barcelona, Paris, and Graz). GWAS analyses were run using PLINK2 [20]; X chromosome data was analyzed by coding male genotypes as haploid and adjusting for sex. Fixed effects meta-analysis was performed with METAL to combine results across the subsets [21]. The primary GWAS of AMS treatment response was performed using the combined AMS A score (i.e. all AMS drugs in the dataset) as the outcome excluding individuals with a B score greater than 4. GWAS were also performed for VPA and LTG separately to assess medication-specific pharmacogenomic effects. All GWASs were adjusted for age, sex, PCs for ancestry, and genotyping batch if needed (full covariate list for each analyzed subset is provided in Table S1). Forest plots were generated to show results across the five subsets of the data for all genetic variants reaching genome-wide significance and for significant variants reported in the previously published GWAS of AMS response in BD [13]. The GWAS summary statistics were used to perform MAGMA gene- and pathway-based analyses using FUMA [22], mapping SNPs to genes if within 50 kb of the gene region. Statistical significance for SNP-level association was set at *p* < 5E-08 (genome-wide significance threshold), while that for gene-level association was set at the Bonferroni-corrected threshold of *p* < 2.5E-06 (19740 protein-coding genes).

### Polygenic score analyses

Polygenic score (PGS) analyses were used to (1) evaluate the polygenic contribution to AMS treatment response across the MoStGen subsets and (2) test whether the genetic liabilities for selected neuropsychiatric disorders are associated with AMS treatment response.

1. For the former, a PGS for AMS treatment response was calculated in each subset of the data using a “leave-one-out” (LOO) approach. For example, the PGS for AMS treatment response for Halifax was estimated using GWAS summary statistics from a meta-analysis of only MCBDB and CBPG subsets, and excluding Halifax from the GWAS training sample. LOO-PGS were calculated using either the AMS combined GWAS or the medication-specific (VPA or LTG) GWASs. Because of the small sample size of the AMS treatment response GWAS, PGS could not be estimated using an auto-tuning approach such as LDpred2 [23]. Instead, we estimated the LOO-PGS using the PRS-PCA approach [24], which combines PGS across multiple *p*-value thresholds (*p*_T_ < 0.0001, 0.001, 0.01, 0.05, 0.1, and 1) computed using PRSice2 into a single PGS [25].
2. To test whether the genetic liabilities for selected neuropsychiatric disorders are associated with AMS treatment response, PGSs were calculated using GWAS summary statistics for attention-deficit/hyperactivity disorder (ADHD; [26]), BD [27], major depressive disorder (MDD; [28]), anxiety disorders [29], schizophrenia [30], and generalized epilepsy [31]. These PGS were estimated using LDpred2-auto [23].

Linear regression was used to test for association of all PGS described above with AMS treatment response within each MoStGen subset using the same covariates as in the GWAS. PGS association results were combined across subsets using fixed effects meta-analysis with the *meta* package in R.

## Results

### Subject characteristics

A summary of participant clinical characteristics is shown in Table 1. As shown in Table S2, participant characteristics varied across sites with respect to sex, age at enrollment, BD type, and ancestry. The MCBDB-Mayo and Halifax datasets had the highest percentages of males ( ~ 45%) while MCBDB2 had the lowest percentage ( ~ 27%; Fig. S1A). Participants in the Halifax and CBPG studies were slightly older at enrollment (mean ages of 47.8 and 47.0 years, respectively) than those from the MCBDB (mean age of 40.7 years; Fig. S1B). The MCBDB1 dataset consisted of a high percentage of patients with BD-I (83.8%) in contrast to other datasets (56.8% to 72.4%; Fig. S1C). European ancestry was the majority in all datasets (71.0% to 98.9%). Latin American ancestry was more prevalent in MCBDB2 as this dataset contained participants recruited in Mexico and Chile (22.8%), but was rare in other datasets (0% to 2.4%). African (2.73%), East Asian (0.65%), and South Asian (0.76%) ancestries had low representation across all available datasets (Fig. S1D).

We observed significant differences in A_ex_ scores for combined AMS (Kruskal-Wallis test: *χ*^2^ = 31.49, *p* < 0.001) and VPA (Kruskal-Wallis test: *χ*^2^ = 44.83, *p* < 0.001) among contributing cohorts, but not for LTG (Fig. S1E). Overall AMS and VPA A_ex_ scores were higher in MCBDB-Mayo compared to all other datasets (Fig. S1E). The VPA A_ex_ scores of MCBDB1 were significantly lower than most datasets apart from MCBDB2. The average Alda scale scores of each site are listed in Table S2 and the combinations of AMS for the calculation of overall AMS A score are shown in Table S3. The distributions of each Alda B scale subitem score by medication are shown in Table S4.

**Fig. 1:**
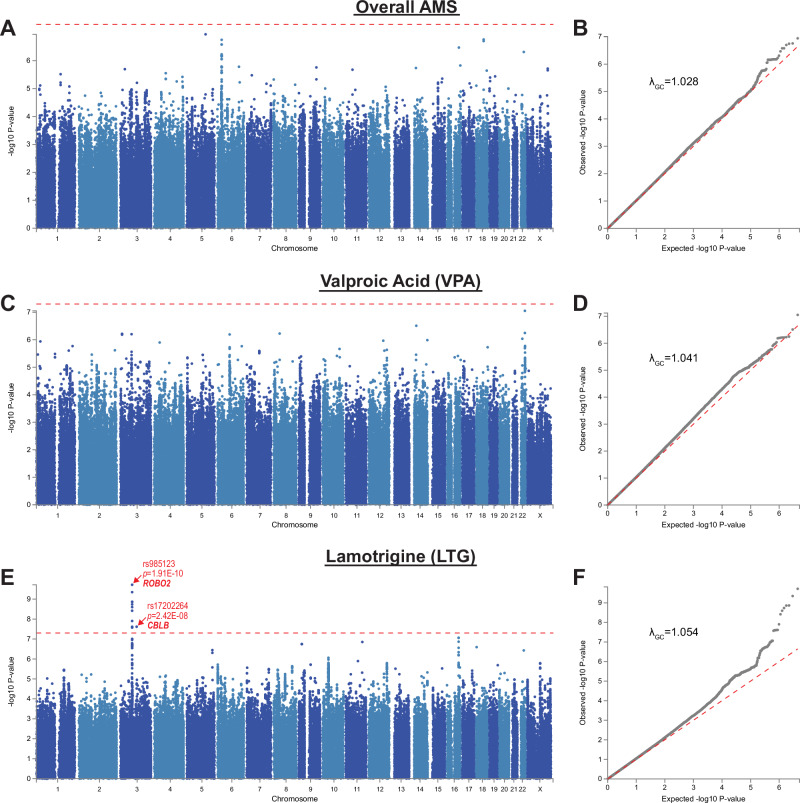
Manhattan and Q-Q plots of the meta-analyses of GWAS of AMS treatment response based on Alda scale A score excluding subjects with B sum score greater than 4. (**A** and **B**) Treatment response on any antiepileptic drug mood stabilizers (AMS); (**C** and **D**) treatment response on valproic acid; (**E** and **F**) treatment response on lamotrigine. The genome-wide significance threshold *p* = 5E-08 is denoted by the red dotted line in the Manhattan plots.

### GWAS of AMS treatment response

No significant SNP-level or gene-level associations were observed for the overall AMS A_ex_ score (i.e. all AMS combined; Fig. 1A and Fig. S2A; top results in Table S5). Similarly, no significant SNP-level or gene-level associations were found in the VPA A_ex_ score analysis (Fig. 1C and Fig. S2C, respectively; top results in Table S6). In the GWAS of LTG A_ex_ score, a genetic locus located on chromosome 3 that maps to a region surrounding exon 3 of *ROBO2* contained 12 SNPs that attained genome-wide significance (*p* = 1.91E-10 to 2.67E-08, *I*^2^ = 0% for all, *p*_het_ = 0.63 to 0.87; Fig. 1E). Fig. S3 shows the association of lamotrigine response (LTG A_ex_) with the top SNP, rs985123, in each cohort. This SNP as well as the neighboring SNPs rs149208967 (*p* = 4.48E-10) and rs140439865 (*p* = 1.25E-08), are near a predicted active transcription start site at intron 1 that may be involved in transcription regulation of this gene and its isoforms in various adult human brain regions (Fig. S4). Another SNP that constituted the signal peak, rs141514083 (*p* = 2.82E-07), is located in a predicted open chromatin region that matches several transcription factor motifs (Fig. S4). Genome-wide significance was also detected for a SNP on chromosome 3 that mapped to *CBLB*, but the association with this SNP showed a notable degree of heterogeneity across datasets (rs17202264: *p* = 2.42E-08, *I*^2^ = 60%, *p*_het_ = 0.056; Fig. 1E), suggesting this association is less robust. The results for *ROBO2* were based on the three MCBDB subsets and Halifax data, as these SNPs were excluded from analysis of the CBPG dataset due to low MAF (Table 2 and Table S7). In the gene-level analyses, one gene, *POLR1E*, was found to be significantly associated with LTG A_ex_ score (*p* = 2.53E-06; Fig. S2E).

**Table 2:** Top SNPs in the meta-analyses of GWAS on LTG treatment response by Alda A score excluding B sum score > 4 (A_ex_ score).

SNP ID	Chr	Position	Nearest Gene	Location in Gene	Effect Allele (A1)	A1 Allele Frequency Range	*β*	*SE*	*dosR*^2^^	*p*	Heterogeneity of effects			
											Direction*	*I*^*2*^ (%)	χ^2^	*p*_het_
rs985123	3	76309081	*ROBO2*	intronic	T	0.016 – 0.055	-3.234	0.508	0.98	1.91E-10	- ? - - -	0	1.378	0.711
rs149208967	3	76322486	*ROBO2*	intronic	A	0.015 – 0.059	-3.153	0.506	0.97	4.48E-10	- ? - - -	0	1.743	0.627
rs75359811	3	76415532	*ROBO2*	intronic	A	0.016 – 0.062	-2.956	0.488	0.99	1.36E-09	- ? - - -	0	0.823	0.844
rs143313661	3	76413390	*ROBO2*	intronic	G	0.016 – 0.062	-2.956	0.488	0.99	1.37E-09	- ? - - -	0	0.823	0.844
rs17735925	3	76427293	*ROBO2*	intronic	G	0.016 – 0.062	-2.951	0.491	0.98	1.83E-09	- ? - - -	0	0.821	0.844
rs17736190	3	76433724	*ROBO2*	intronic	T	0.019 – 0.062	-2.884	0.484	0.98	2.52E-09	- ? - - -	0	1.076	0.783
rs141943567	3	76298949	*ROBO2*	intronic	A	0.016 – 0.055	-3.118	0.529	0.97	3.83E-09	- ? - - -	0	0.931	0.818
rs140439865	3	76315449	*ROBO2*	intronic	T	0.016 – 0.062	-2.895	0.509	0.98	1.25E-08	- ? - - -	0	1.471	0.689
rs17202264	3	105698103	*CBLB*	intronic	C	0.014 – 0.045	-3.750	0.672	0.77	2.42E-08	- - - - ?	60.4	7.581	0.056
rs1878209	3	76255981	*ROBO2*	intronic	A	0.013 – 0.055	-2.720	0.488	0.97	2.44E-08	- ? - - -	0	1.757	0.624
rs148781824	3	76295321	*ROBO2*	intronic	C	0.013 – 0.055	-2.976	0.534	0.98	2.49E-08	- ? - - -	0	0.735	0.865
rs1516474	3	76275736	*ROBO2*	intronic	T	0.014 – 0.055	-2.975	0.534	0.98	2.50E-08	- ? - - -	0	0.735	0.865
rs149261613	3	76260751	*ROBO2*	intronic	A	0.014 – 0.055	-2.977	0.535	0.97	2.67E-08	- ? - - -	0	0.727	0.867

*Directions of the associations of the Effect Allele with the outcome across GWAS datasets (positive + or negative -). Datasets from left to right: Halifax, CBPG, MCBDB1, MCBDB2, and MCBDB-Mayo. Question mark indicates association was not estimated. ^ dosR^2^: Average imputation quality across the 5 cohorts as measured by dosage R^2^.

### Analysis of previously identified genetic variants

The top SNPs previously reported to be associated with AMS treatment response [13], including *THSD7A* rs78835388, *ABCC1* rs875740, *DISP1* rs34701716, and *VAV3* rs13353016, were not associated with AMS A_ex_ score at a genome-wide significant level; while each of the four SNPs showed some trends of association in the MoStGen Consortium meta-analyses (Bonferroni-adjusted *p* < 0.0125 for four SNPs; Fig. S5), this was mostly due to samples that were included in the original publication (subset of MCBDB1 and MCBDB-Mayo).

### Pathway enrichment analysis

No significantly enriched pathways were detected in the GWAS of combined AMS, VPA, and LTG treatment response (Bonferroni-corrected *p* > 0.05; Table S8).

### Leave-one-out (LOO) PGS analysis of AMS treatment responses

**Fig. 2:**
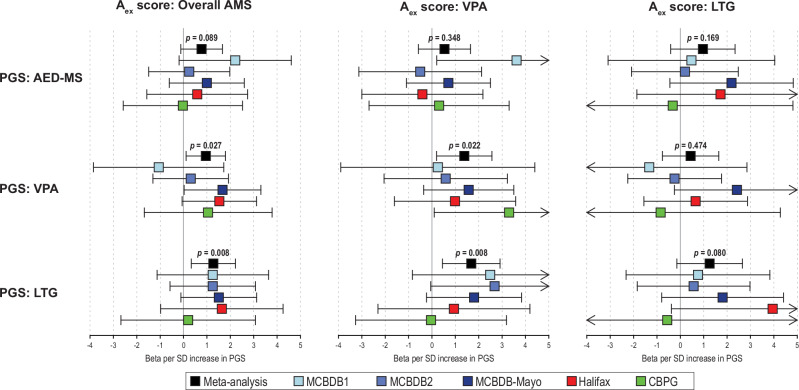
Leave-one-out analysis of the associations between the polygenic scores (PGS) of all AMS, valproic acid (VPA), and lamotrigine (LTG) treatment response and the Alda A scores excluding subjects with B score > 4 (A_ex_ scores) for all AMS (left panel), VPA (middle panel), and LTG (right panel). The meta-analysis *p*-value between a PRS and an A_ex_ score is shown above each meta-analysis bar (i.e., black box). There was no heterogeneity of effects across sites for all associations tested (*I*^2^ = 0).

The LOO-PGS using results from the combined GWAS of all AMS was positively correlated with the overall AMS A_ex_ score but the association was not statistically significant (Fig. 2). However, when using LOO-PGSs corresponding to the medication-specific GWAS of VPA and LTG treatment response, both LOO-PGSs were significantly associated with overall higher AMS A_ex_ score (VPA: *β* = 0.95 increase in A score per 1 SD increase in PGS, 95%CI = 0.11 – 1.79, *p* = 0.027; LTG: *β* = 1.28, 95%CI = 0.33 – 2.22, *p* = 0.008; Fig. 2). The observed PGS associations were largely consistent across datasets (Fig. 2) and demonstrated no evidence of heterogeneity of effects across subcohorts (*I*^2^ = 0). Detailed LOO-PGS results, including the drug-stratified analyses, are presented in Table S9.

### AMS treatment response associations with the PGSs of epilepsy and psychiatric illnesses

**Fig. 3:**
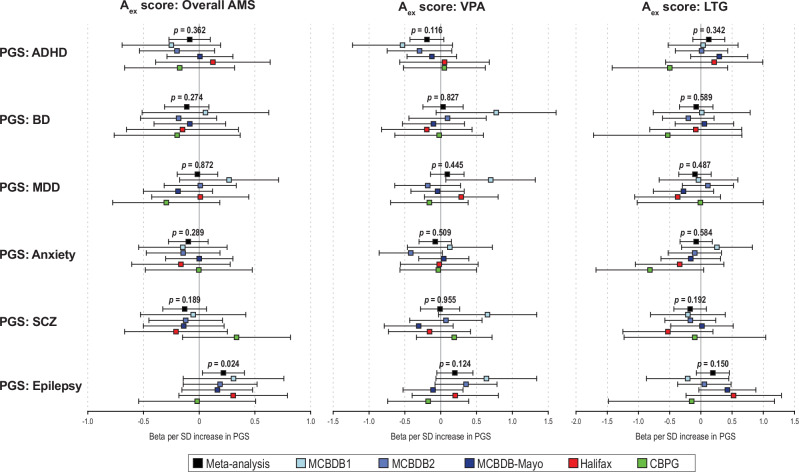
Associations between the polygenic scores (PGS) of various neuropsychiatric illnesses and the Alda A scores excluding subjects with B score > 4 (A_ex_ score) for all AMS (left panel), valproic acid (VPA; middle panel), and lamotrigine (LTG; right panel). The meta-analysis *p*-value between a PRS and an A_ex_ score is shown above each meta-analysis bar (i.e., black box). ADHD: attention-deficit/hyperactivity disorder; BD: bipolar disorder; MDD: major depressive disorder; SCZ: schizophrenia. There was no heterogeneity of effects across sites for all associations tested (*I*^2^ = 0).

While none of the psychiatric PGSs were significantly associated with overall AMS A_ex_ scores, the generalized epilepsy PGS showed suggestive evidence of association with higher overall AMS A_ex_ scores (*β* = 0.217, 95%CI = 0.03 – 0.041, *p* = 0.024, *I*^2^ = 0; Fig. 3), although this result would not remain significant after multiple testing correction. Detailed LOO-PGS results are presented in Table S10.

## Discussion

The MoStGen Consortium was established to enable well-powered studies of potential pharmacogenomic markers and clinical predictors of AMS treatment response in BD. This international collaboration has so far accumulated AMS treatment response and genomic data from nearly 1000 BD patients. Our meta-analysis across multiple sites with the Alda A score (excluding those with B score >4) as the outcome did not replicate association findings reported previously [13]. Instead, we detected a strong genome-wide significant association with LTG response that mapped to *ROBO2*. Leave-one-out PGS analysis provided evidence of polygenic contributions to AMS treatment response, which were strengthened when considering polygenic signal specific to VPA or LTG treatment response. While most of the examined PGSs for neuropsychiatric illnesses were not predictive of AMS treatment response, a higher PGS for generalized epilepsy was significantly associated with better AMS response.

A genetic locus on chromosome 3 that maps to *ROBO2* showed significant association with LTG treatment response. *ROBO2* encodes roundabout guidance receptor 2, which is crucial in axon guidance, dendrite development, neural progenitor cell proliferation, neurogenesis, and cortical neuron migration [32–35]. Apart from the developing brain, ROBO2 also plays a vital role in adult brain function, impacting spatial memory and cognitive tasks [36], as evidenced by its association with dorsolateral prefrontal cortex activity in schizophrenia patients [37]. Moreover, *ROBO2* has been implicated in dyslexia [38] and autism spectrum disorder [39, 40]. ROBO2 has also been identified as one of the plasma proteins that were downregulated in patients with schizophrenia and BD who were not on antipsychotics but were normalized in those who were on antipsychotics [41]. However, its relationship with the improvement of mood symptoms, and whether such change is specific to certain BD pharmacotherapeutics, requires further investigation. While the top SNPs associated with LTG treatment response in *ROBO2* were not eQTLs, their proximity to active chromosome state regulatory sites (Fig. S4) suggests potential regulatory roles in gene transcription. The association of *POLR1E* (RNA polymerase I subunit 1E) with BD remains relatively unexplored, and its gene expression was not detected in human brain cells (Fig. S6), but its involvement in epilepsy treatment has been noted [42].

In a prior study of AMS treatment response, we reported associations with *THSD7A* rs78835388 and *SLC35F3* rs114872993 at SNP-level, and with *ABCC1* and *DISP1* at gene-level [13]. Although some of these top SNPs were among those most statistically significant in the MCBDB-Mayo dataset, which included subjects that were in the previously published GWAS, we were unable to replicate these findings with the larger sample contributing to the current meta-analysis. Such irreproducibility may be attributed to the “winner’s curse” [43], which can be prominent in low-power scenarios, as well as to differences between the two studies. First, to maximize data use from a small sample, the previously published analysis used the total Alda score (i.e., A score minus B sum score) as the treatment response. In the present larger study, the A_ex_ score was used as the outcome, which has been found to be more reliable than the total score and the A score without the consideration of the B sum score [9, 14], analysis of the total Alda score in the current MoStGen sample also did not replicate the prior finding. Second, the genetic ancestry composition of the datasets in this study was more diverse compared to the previous study, which consisted solely of participants of European ancestry. However, the proportion of patients of non-European ancestries in this study was also small (Fig. S1D), and analyses excluding non-European ancestries from this study did not improve replication (data not shown). Third, the previous study only included BD patients recruited in the US, while the MoStGen Consortium includes multiple international groups; thus differences in pharmacotherapeutic patterns according to corresponding countries’ prescription practices are expected [44]. For example, lithium is more commonly prescribed in Europe than in North America, which may be due to multiple factors such as the prescriber’s clinical experience, the financial burden of monitoring laboratory tests on patients, and prescriber’s concern for the potential development of renal insufficiency and thyroid dysfunction [44]. Such prescription differences may lead to uneven selection bias of patients taking a given medication across sites, as in some countries an AMS may not be used as the initial treatment and is only prescribed when patients need to switch medications (e.g., unresponsive to current medications, intolerable side effects, change in the course of illness). Thus, patients with AMS treatment response data may differ in systematic ways across sites, representing a population with greater treatment resistance for some sites. Such heterogeneity among samples may have hindered the replication of pharmacogenomic association findings.

This study demonstrated a polygenic signal of AMS treatment response. Indeed, while the PGS explained a very small percentage of the outcome variance ( < 1%), this is the first study that reports significant polygenic effects for overall AMS treatment response and for medication-specific treatment response (VPA and LTG) in BD. Additionally, we observed a suggestive positive association between epilepsy PGS and overall AMS A_ex_ score, indicating that patients with a greater genetic burden of epilepsy may experience more effective mood-stabilizing efficacy with AMSs. This result also implies that certain genetic factors underlying epilepsy may also contribute to mood instability in BD, as previously suggested [45]. Some recent studies found significant genetic correlations between epilepsy and BD, but the direction and magnitude of these correlations — as well as the implicated overlapping genetic variants — varied across studies [46–48]. Despite differences in etiologies and symptom profiles, AMSs are used in both epilepsy and BD prophylaxis. Major AMSs such as VPA and LTG share some mechanisms of action by blocking voltage-gated sodium and calcium channels [49, 50]. Overall, our PGS findings should be interpreted cautiously, and further research is needed to elucidate the shared biological and therapeutic mechanisms underlying epilepsy and BD.

Apart from the aforementioned heterogeneity among datasets, as well as challenges of meta-analysis such as differential missingness of SNP data across datasets due to differences in genotyping platforms or allele frequences across cohorts, this study had other limitations. While we recognize that various AMSs have distinct pharmacological properties, proposed mechanisms of action, and specific efficacy, they were grouped for the “combined AMS” analyses due to their commonality in possessing anticonvulsive and mood-stabilizing effects, as well as to maximize sample size. Nonetheless, this study also included the first VPA- and LTG-specific GWAS, which are expected to provide pharmacogenomic insights specific to these medications. The sample consisted mostly of patients of European ancestry (88.2%), limiting the potential generalizability of the results due to potential differences in AMS pharmacokinetics and pharmacodynamics among ancestry groups. Finally, although we used B sum > 4 as a criterion to exclude A scores that were unlikely to be attributed to the medications of interest, factors measured by the individual B subscores may affect treatment outcomes differently, hence their distinct relationships with both genetics and overall treatment response should be explored further in larger samples [51, 52].

In conclusion, the MoStGen Consortium was established to identify pharmacogenomic markers for AMS treatment response in patients with BD. Using this international dataset, we identified a novel genetic locus in *ROBO2* that is associated with LTG treatment response in BD. Furthermore, we demonstrated a polygenic signal for AMS treatment response and that a greater genetic burden for epilepsy might predict a better treatment response to AMS in patients with BD. These findings should be considered exploratory and warrant replication in other datasets as well as validation in cohorts with non-European ancestries. Nevertheless, they may lead to new insights into AMS mood-stabilizing properties and present potential markers for treatment response. Overall, this study discovered novel potential genetic contributions to AMS treatment response, which is another step toward personalized medicine for BD.

## Supplementary information

Supplementary Methods & Figures

Supplementary Tables
