# Supplementary material for: Pharmacogenomics of antiepileptic drug mood stabilizer treatment response in bipolar disorder: A MoStGen Consortium study
Source: Mol Psychiatry. 2026 Feb 19;31(6):3587–94. doi: 10.1038/s41380-026-03478-7 (PMC13190236; doi:10.1038/s41380-026-03478-7)
Supplement: Supplementary file 1 — Supplementary Methods & Figures [file 41380_2026_3478_MOESM1_ESM.pdf]

Supplementary Materials

Pharmacogenomics of antiepileptic drugs mood stabilizer treatment response in bipolar disorder patients: a MoStGen Consortium study

Table of Contents

Supplementary Methods **2**
Individual Sample Descriptions 2
Supplementary Figures **5**
Fig. S1. Distributions of sex, age, diagnosis, ethnicity, and Alda A_{ex} scores of the five GWAS subsets.......... 5
Fig. S2. Manhattan and Q-Q plots of gene-level GWAS 6
Fig. S3. *ROBO2* top SNP association with LTG treatment response by cohorts 7
Fig. S4. Locus zoom plot of top SNPs in *ROBO2* and key predicted regulatory features in the region. 8
Fig. S5. Validation of previously reported top SNPs associated with AMS treatment response 9
Fig. S6. Gene expression patterns of *ROBO2, CNTNAP4* and *POLR1E* in human brain cells 10
References **11**

Supplementary Methods

Sample Descriptions

Mayo Clinic Bipolar Disorder Biobank (MCBDB), U.S.A./Mexico/Chile | Joanna Biernacka, Mark Frye, Susan McElory, Alfredo Cuellar, Miguel Prieto

The Mayo Clinic Bipolar Disorder Biobank is a multi-site collaboration centralized at the Mayo Clinic [1]. In the U.S., participants were recruited and enrolled at the Mayo Clinic, Rochester, Minnesota; Lindner Center of HOPE/University of Cincinnati College of Medicine, Cincinnati, Ohio; and the University of Minnesota, Minneapolis, Minnesota. The Biobank also recruited participants at Universidad Autonoma de Nuevo León in Mexico as well as Universidad de los Andes in Chile. Participants were identified through routine clinical appointments, from in-patients admitted in mood disorder units, and recruitment advertising. Participants were required to be between 18 and 80 years old, be able to speak English or Spanish (in Mexico and Chile), provide informed consent, and have a confirmed DSM-IV-TR diagnosis of type 1 or 2 bipolar disorder or schizoaffective bipolar disorder as determined by the Structured Clinical Interview for DSM-IV (SCID)[2]. Enrolment procedures and protocols were approved by local Institutional Review Boards and all participants provided written informed consent for their participation in the biobank and the use of their data in future genetic studies. Genotyping was conducted in three waves since 2012, as DNA samples accumulated to sizable batches suitable for genotyping by the latest technologies at the time. The first two batches (MCBDB1 and MCBDB2) were genotyped using Illumina microarrays, while genotyping data from exome sequencing were obtained for the latest batch (MCBDB-Mayo; **Supplementary Table S1**). Exome sequencing with Genotype-by-Sequencing (GxS) technology was performed at the Regeneron Genetics Center (Tarrytown, NY, U.S.A.). This technology captures the “backbone” regions of the genome at lower depths than exome regions with genotyping quality enhanced after data post-processing [3].

Halifax and Ottawa, Canada | Martin Alda

The case samples were recruited from patients longitudinally followed at specialty mood disorders clinics in Halifax and Ottawa (Canada). Cases were interviewed in a blind fashion with the Schedule of Affective Disorders and Schizophrenia-Lifetime version (SADS-L) [4] and consensus diagnoses were made according to DSM-IV [5] and Research Diagnostic Criteria (RDC) [6]. Protocols and procedures were approved by the local Ethics Committees and written informed consent was obtained from all patients before participation in the study. The evaluation of treatment response has been performed using all available information including diagnostic interviews, medical records, and data from longfitudoinal follow-up. The investigators were all trained in the use of the ALDA scale and evaluated for their inter-rater reliability [7].

Cagliari, Italy | Mirko Manchia

Participants were recruited consecutively at the Psychiatric Unit of the University Hospital of Cagliari and of the Department of Medical Sciences and Public Health, University of Cagliari. The study was approved by the local ethics committee (Ethics Committee of the University Hospital Agency of Cagliari: PG/2019/6277 and subsequent amendments) in compliance with the current revision of the Declaration of Helsinki and the current EU-regulations for the protection of privacy. The following inclusion criteria were applied for the population of patients with BD: a) presence of diagnosis of BD type 1 or type 2 according to the DSM-5 criteria; b) age between 18 and 65 years old. Diagnoses and clinical assessments

were performed by trained psychiatrists through direct interview and a systematic review of patients' medical records. The diagnosis of BD was confirmed with the Italian version of the Structured Clinical Interview (SCID) according to DSM-5. Individuals were excluded if: a) they were women in the fertile age, who did not use adequate contraception or who were pregnant; b) they had a history of traumatic brain insults; c) they had a diagnosis of current and/or lifetime other psychiatric or neurological disorders or other severe unregulated medical conditions; d) they had a diagnosis of current and/or lifetime substance use disorder. The assessments included the collection of sociodemographic parameters, illness history (including the number and entity of illness episodes), psychotic features, suicide attempts, medication history, current psychiatric symptomatology measured with validated rating scales, and treatment response using the Retrospective Criteria of Long-Term Treatment Response in Research Subjects with Bipolar Disorder (Alda scale) scale.

Specifically, we performed a detailed revision of the clinical charts to permit a graphic depiction of the longitudinal clinical course with the life chart method. In our study, this included both the retrospective assessment of past clinical courses (based on accurate longitudinally collected clinical data) and prospective three-year observation. Then, we calculated the area under the curve of illness activity (severity of the episodes x duration of episodes) before and after the introduction of a mood stabilizer or combination of mood stabilizers. This gave us an objective measure of clinical improvement under a specific treatment or combination of treatments (criterion A of the Alda's scale). The assessment of clinical response to mood stabilizers was performed by trained psychiatrists with the supervision of one senior rater (M. M.) who has worked in the validation procedure of the scale [8]. In this naturalistic study, some patients took combinations of mood stabilizers for variable duration or were treated sequentially with diverse mood stabilizers. Here we are expressing the improvement observed under each mood stabilizer and not concomitant treatment.

Paris, France | Marie-Odile Krebs, Boris Chaumette
We recruited participants as part of the PSYDEV collection ("Etude familiale et génétique des aspects développementaux des maladies psychiatriques"), a study based in Paris, France, that recruits both inpatients and outpatients. Participants were assessed for capacity to provide informed consent by the clinical team. All participants provided written informed consent. Oral consent was asked for individuals under the age of 18 or under guardianship but the written consent was obtained from their legal representatives. The study had ethics approval granted by CPP Ile de France IV which permits inclusion of the data in large international studies.

Participants were interviewed by board-accredited psychiatrists and kept if they met the criteria for bipolar disorder. Trained raters reviewed this interview, along with available clinical records, to determine a consensus lifetime DSM-IV diagnosis. Response to treatment was recorded using the Alda scale. The recruitment was funded by INSERM U1266 (Institute of Psychiatry and Neurosciences of Paris) and Sainte-Anne Hospital (GHU Paris Psychiatrie & Neurosciences).

Blood was sampled by venous puncture. Samples were genotyped on the Illumina GSA at the McGill Genome Center (Montreal, Canada). The genotyping was funded by the ERAPerMed Plot-BD grant.

Barcelona, Reus and Sant Boi, Spain | Eduard Vieta
Cases were recruited from the Bipolar Disorder Program of the University of Barcelona and Hospital Clinic, Pere Mata Hospital, and FIDMAG, under the umbrella of the Spanish Research Network on Mental Health (CIBERSAM). Participants were selected only if they fulfilled the following inclusion

criteria: (i) met DSM-IV-TR criteria for BD-I or -II, (ii) age over 18 years, (iii) met criteria for euthymia for at least 3 months before inclusion, assessed by the Hamilton Depression Rating Scale (HDRS) and the Young Mania Rating Scale (YMRS), and (iv) provided both written and verbal informed consent. Exclusion criteria were as follows: (i) intelligence quotient (IQ) lower than 70, (ii) the presence of any medical condition affecting neuropsychological performance, and (iii) electroconvulsive therapy within the past year. The study was approved by each institution's ethics committee and was performed in accordance with the ethical principles of the Declaration of Helsinki.

Graz, Austria | Susanne Bengesser, Eva Reininghaus

Patients were recruited at the Medical University of Graz, Department of Psychiatry and Psychotherapeutic Medicine. All patients received a lifetime diagnosis of BD according to the DSM-IV criteria on the basis of a consensus best-estimate procedure that considered all available information, including structured diagnostic interviews with the SCID, medical records, and personal medical history. Study protocols were reviewed and approved in advance by the IRBs of the participating institutions. All participants provided written informed consent.

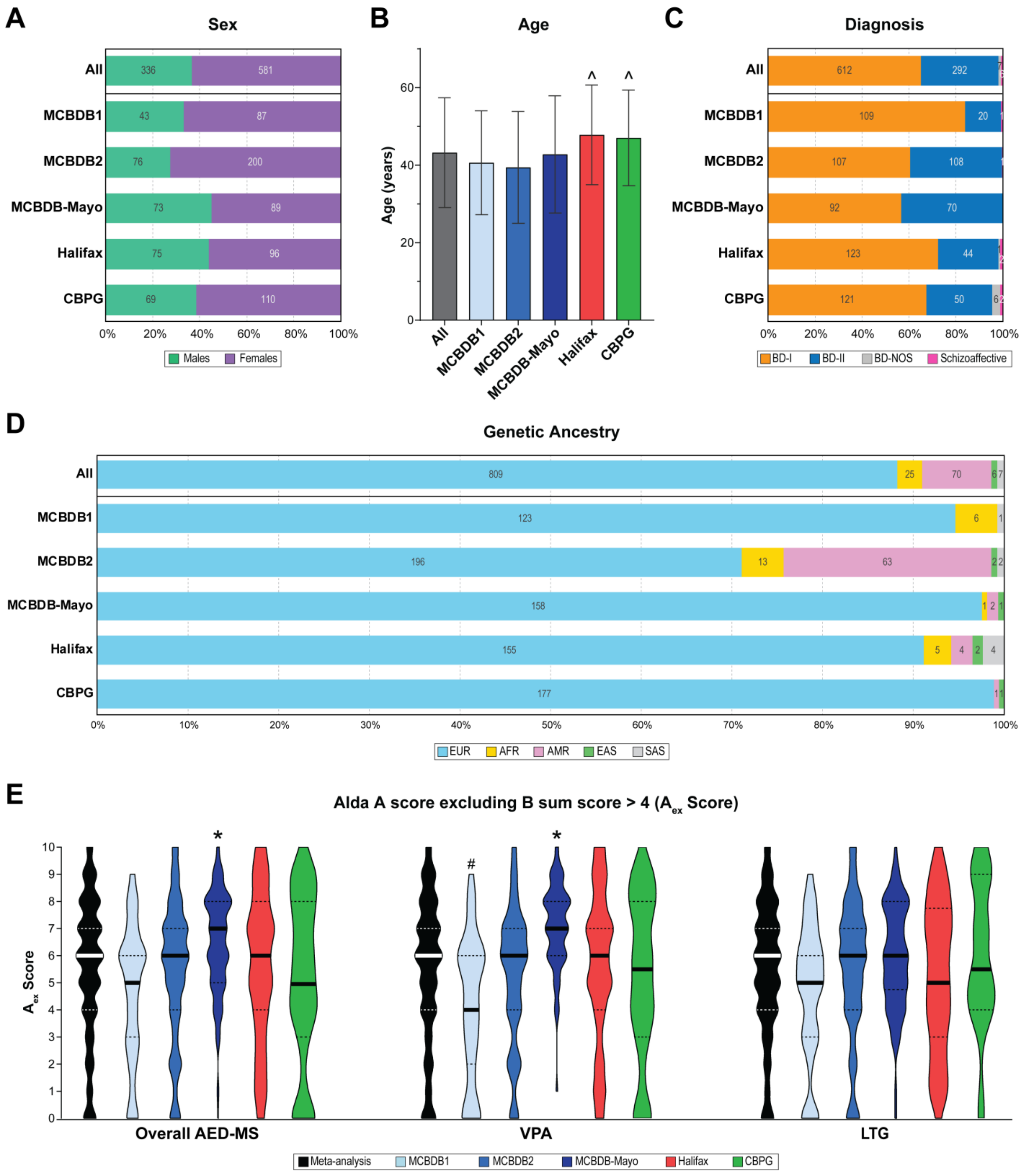

Fig S1. Distributions of (**A**) sex, (**B**) age, (**C**) diagnosis, (**D**) genetic ancestry, and (**E**) Alda A score excluding subjects with B sum score > 4 among subjects (A_{ex} score) of the five GWAS subsets. Median and quartile range are indicated in the violin plots. *Posthoc* $p < 0.05$ when the indicated dataset is compared to all other datasets (*), to all MCBDB sites (^), or to all datasets apart from MCBDB2 (#). BD: bipolar disorder; LTG: lamotrigine; VPA: valproic acid.

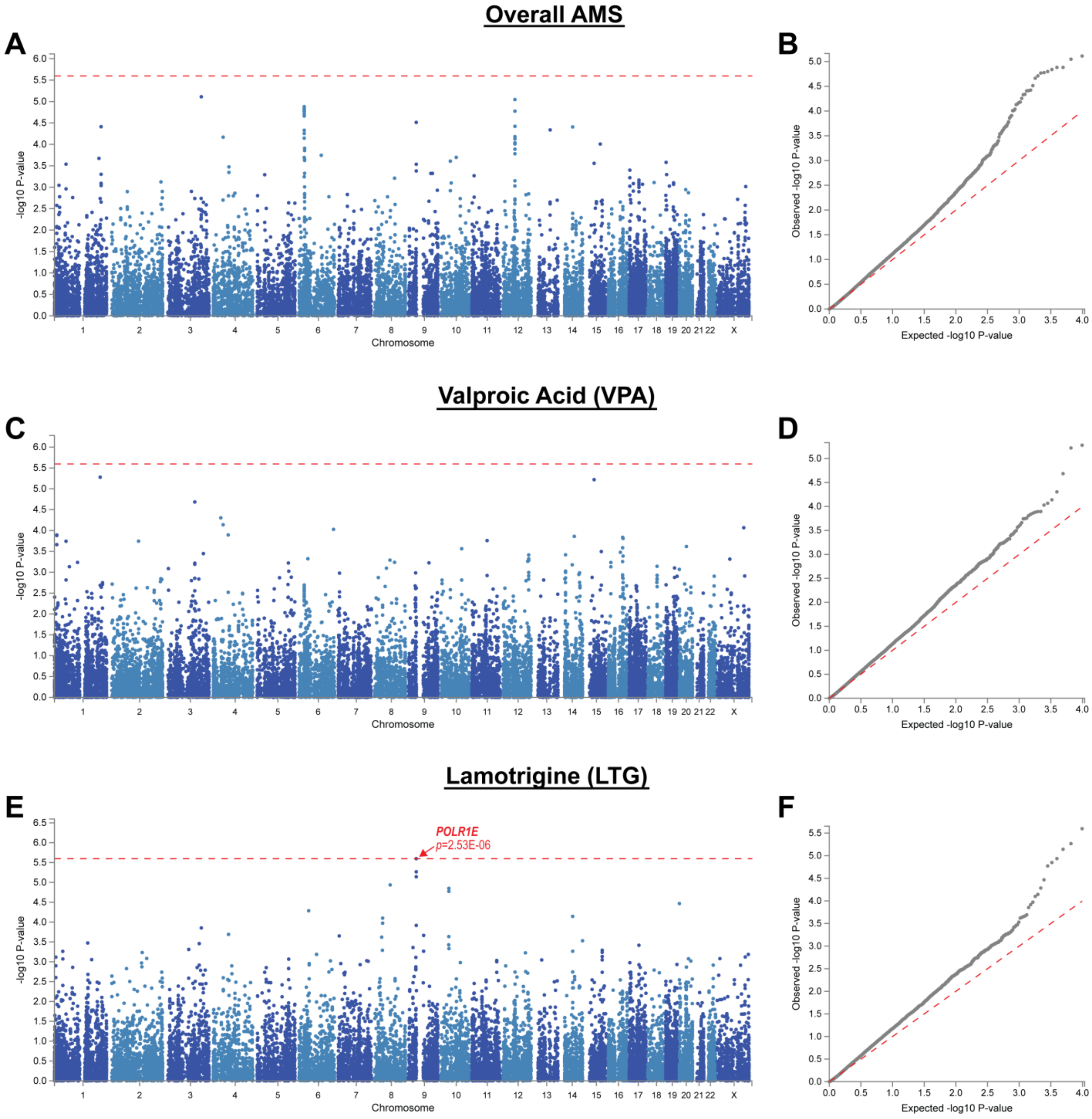

Fig S2. Manhattan and Q-Q plots of the gene-level GWAS analyses of AMS treatment response based on Alda scale A score excluding B sum score > 4. (**A** and **B**) Treatment response for any AMS; (**C** and **D**) valproic acid treatment response; (**E** and **F**) lamotrigine treatment response. The genome-wide gene-level significance threshold p = 2.533E-06 is denoted by the red dotted line in the Manhattan plots.

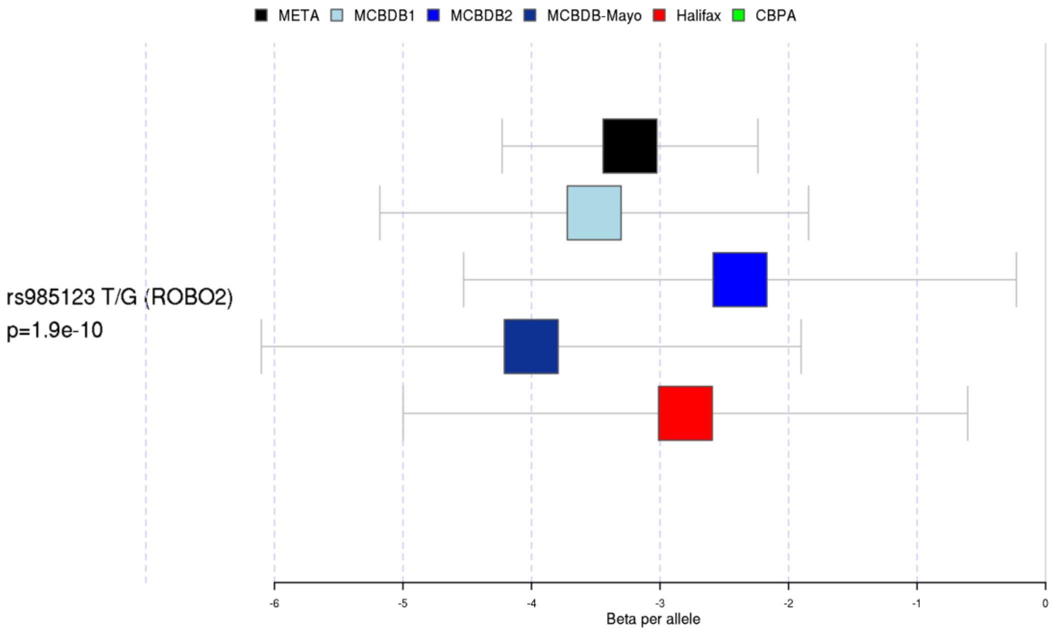

Fig S3. The associations of *ROBO2* top SNP with lamotrigine treatment response by cohorts.

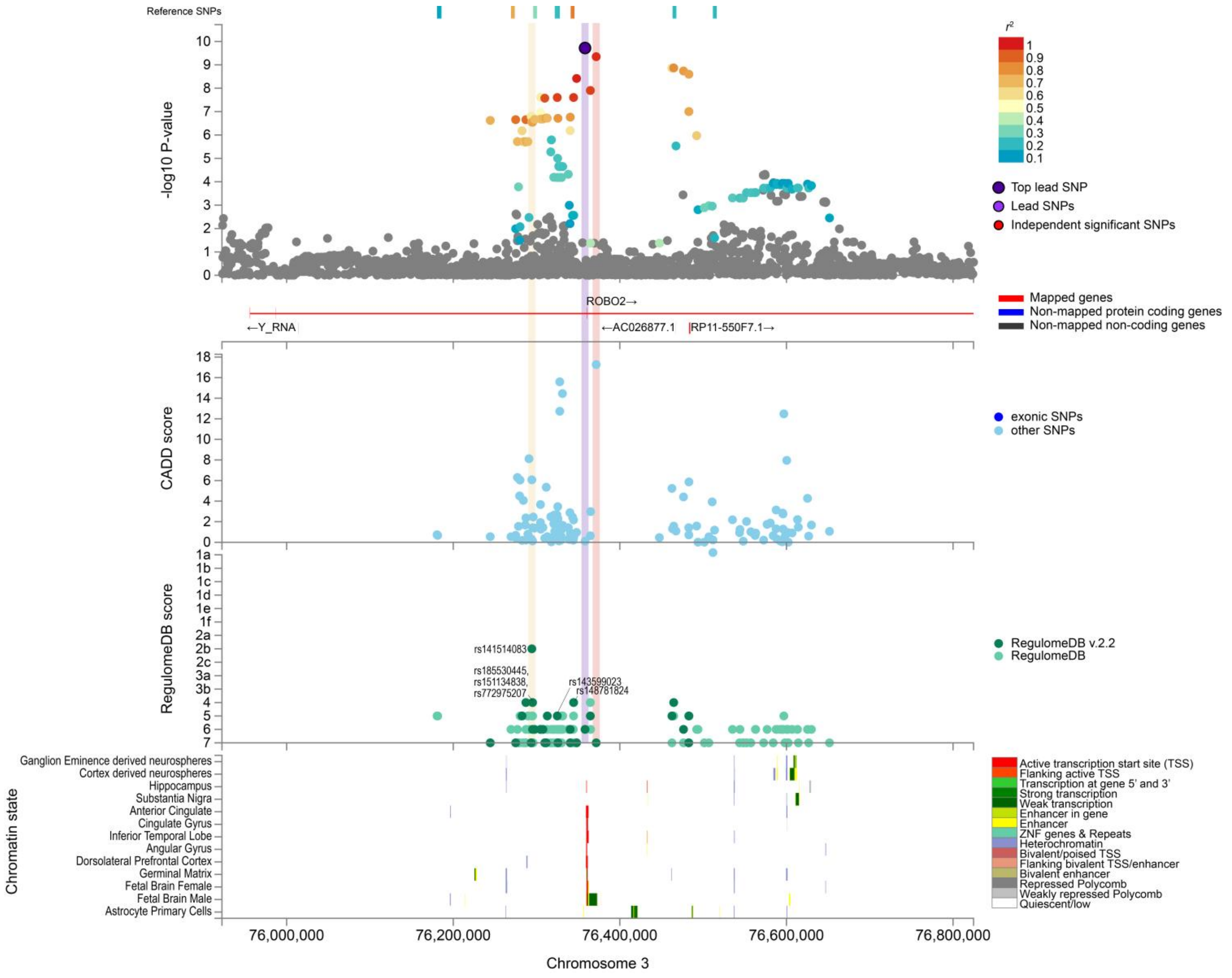

	DNase-Seq/ATAC-Seq	Histone	ChIP-seq	TF Motifs	Chromatin State
rs141514083	Microvascular endothelial cell<br>Choroid plexus epithelial cell		Liver: FOXA2	ARID5A<br>FOXB1<br>FOXC1<br>ONECUT1<br>ONECUT2<br>ONECUT3	
rs185530445,<br>rs151134838,<br>rs772975207		Liver (hepatocyte):<br>H3K27ac	Brain (SK-N-SH): ISL1		
rs143599023			Liver (HepG2): ZNF146	MLXIP<br>NPAS2	
rs148781824			Liver (HepG2): HLF, CEBPB		
rs985123				GFI1B	Choroid plexus epithelial cells: weak transcription
rs140439865					Brain (SK-N-MC): weak transcription<br>Bipolar neuron: weak transcription
rs149208967					Brain (SK-N-MC): weak transcription

Fig S4. Locus zoom plot of the top SNPs in *ROBO2* in the lamotrigine treatment response GWAS. SNPs with $p < 1E\text{-}6$ and notable CADD or RegulomeDB scores (version 2.2; [9, 10]) are highlighted. Regulatory features (focused on the brain and liver) associated with these SNPs are listed in the table below.

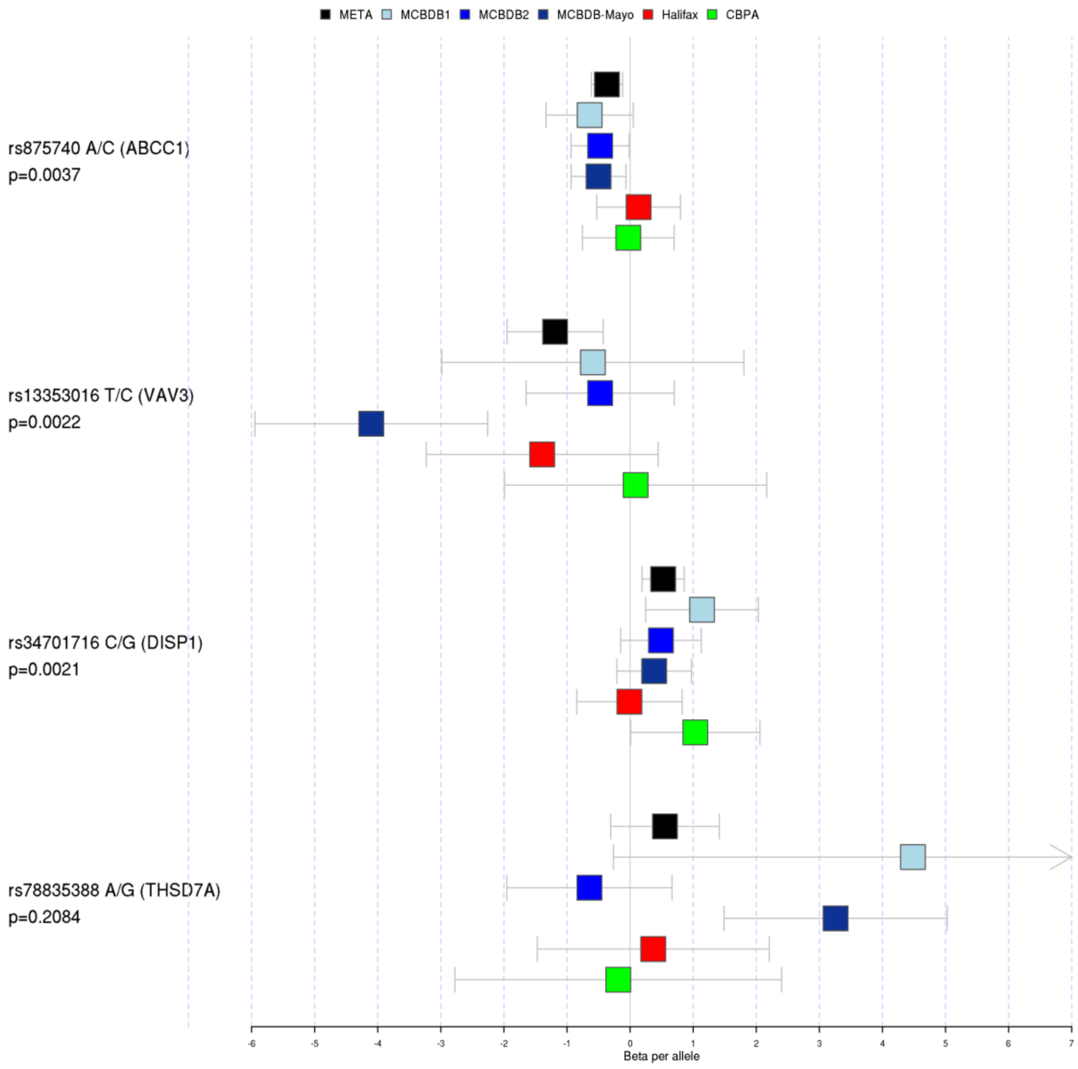

Fig S5. MoStGen Consortium GWAS results for top SNPs associated with AMS treatment response reported in Ho, Coombes *et al.* (2020).

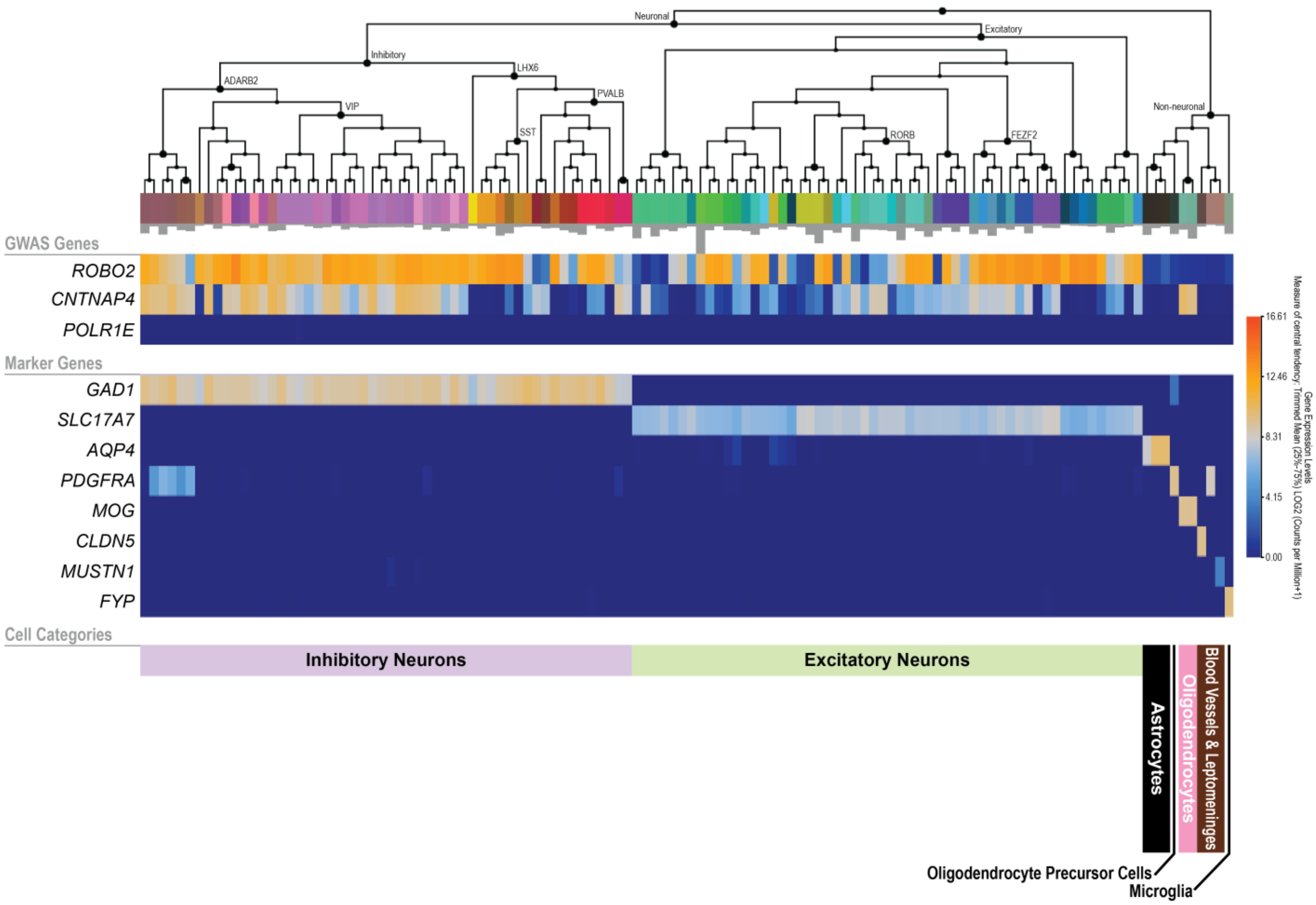

Fig S6. Heatmap of gene expression patterns of genes with notable SNP-level (*ROBO2* and *CNTNAP4*) and gene-level (*POLR1E*) associations with lamotrigine treatment response in human brain cells. RNA sequencing and transcriptomic clustering of single-cell nuclei from six adult human brain cortical regions were conducted by the Allen Institute of Brain Science [11]. The middle panel shows the gene expression patterns of cell type-specific marker genes: *GAD1* (glutamate decarboxylase 1) for inhibitory neurons; *SLC17A7* (vesicular glutamate transporter 1) for excitatory neurons; *AQP4* (aquaporin 4) for astrocytes; *PDGFRA* (platelet-derived growth factor receptor A) for oligodendrocyte precursor cells and vascular and leptomeningeal cell; *MOG* (myelin oligodendrocyte glycoprotein) for oligodendrocytes; *CLDN5* (claudin 5) for microvascular endothelial cells; *MUSTN1* (musculoskeletal embryonic nuclear protein 1) for pericytes; *FYB* (FYN-binding protein) for microglia. The lower panel shows general cell type categories. Increasing levels of gene expression are represented by a color gradient from dark blue to dark orange. The figure shows that *ROBO2* is mostly expressed in neurons (both inhibitory and excitatory neurons). *CNTNAP4* is also expressed in neurons with a preference in inhibitory neurons; it is also expressed in prominently oligodendrocytes. *POLR1E* expression is not detected in all brain cell types.

References

1. Frye MA, McElroy SL, Fuentes M, Sutor B, Schak KM, Galardy CW, Palmer BA, Prieto ML, Kung S, Sola CL, Ryu E, Veldic M, Geske J, Cuellar-Barboza A, Seymour LR, Mori N, Crowe S, Rummans TA, Biernacka JM. (2015) Development of a bipolar disorder biobank: differential phenotyping for subsequent biomarker analyses. *Int J Bipolar Disord.* **3**(1):30.
2. First MB, Gibbon M, Spitzer RL, Williams JBW, Benjamin LS, *Structured clinical interview for DSM-IV axis II personality disorders, (SCID-II)*. 1997, Washington, D.C.: American Psychiatric Press.
3. Gelfman S, Moscati A, Huergo SM, Wang R, Rajagopal V, Parikshak N, Pounraja VK, Chen E, Leblanc M, Hazlewood R, Freudenberg J, Cooper B, Ligocki AJ, Miller CG, Van Zyl T, Weyne J, Romano C, Sagdullaev B, Melander O, Baras A, Zhang A, Mansfield AJ, Locke A, Pandey A, Campos A, Ghosh A, Gorovits A, Lopez A, Hawes A, Fenney A, Averitt A, Joshi A, Damask A, Bunyea A, Ziyatdinov A, Pandit A, Perez-Beals A, Alkelai A, Marcketta A, Baldassari A, Ayer A, Gilly A, Rasool A, Guvenek A, Geraghty B, Sultan B, Palmer W, Ye B, Zhang B, Boutkov B, Hobbs B, Forsythe C, Sidore C, Paulding C, Wang C, Beechert C, Gillies C, Zhang C, Willer CJ, Li D, Sharma D, Stahl E, Austin E, Jorgenson E, Brian ED, Mayerhofer E, Edelstein E, Maxwell EK, Tzoneva G, Hindy G, Mitra G, Solari G, Eom G, Du H, Khiabanian H, Kosmicki J, Otto J, Hernandez J, Clauer J, Mighty J, Staples JC, Rico-Varela J, Brown J, He J, Zhang J, Revez J, Hankins J, Mbatchou J, Rivera-Picart J, Silver J, Bovijn J, Marchini J, Ross J, Bras J, Herman J, Backman J, Guan J, Rodriguez-Flores J, Mower J, Landheer K, Sun K, Burch K, Sosina K, Manoochehri K, Skead K, Punuru KP, Guevara K, Wu K-H, Watanabe K, Zhang L, Cremona LM, Gurski L, Chen L, Ganel L, Sun L, Habegger L, Pradhan M, Kapoor M, Ferreira MAR, Jones MB, Padilla MS, Suciu MC, Ghoussaini M, Haas M, Lattari M, Kessler M, LeBlanc MG, Pagan M, Tang M, Riaz M, Nafde M, Sarwar M, Rana N, Lin N, Verweij N, Banerjee N, Nishtala N, Krasheninina O, Delaneau O, Sosina O, Akbari P, Dornbos P, VandeHaar P, Challa P, Nakka P, Schwartz R, Reynoso R, Panea R, Schiavo R, Guerreiro R, Lanche R, Vedantam S, Martinez SR, Choi S, Zarate S, Malhotra S, Hart S, Sreeram S, Wolf SE, Graham S, Vrieze S, O'Keeffe S, Yu S, Gaynor S, Alvarez S, Balasubramanian S, Gokhale S, Siceron S, Bao S, De T, Thornton T, Polanco T, Joseph T, Zavala V, Kumar V, Mahajan V, Salerno WJ, Bai X, Zou Y, Gu Z, Ferrando A, Shuldiner A, Deubler A, Economides A, Abecasis GR, Reid JG, Overton JD, Siminovitch K, Lotta LA, Mitnaul LJ, Cantor M, Stahl EA, Coppola G, Regeneron Genetics C. (2023) A large meta-analysis identifies genes associated with anterior uveitis. *Nature Communications.* **14**(1):7300.
4. Endicott J, Spitzer RL. (1978) A diagnostic interview: the schedule for affective disorders and schizophrenia. *Arch Gen Psychiatry.* **35**(7):837-44.
5. Association AP, *Diagnostic and statistical manual of mental disorders: DSM-IV.* 1994, Washington, D.C.: American Psychiatric Press, Inc.
6. Spitzer RL, Endicott J, Robins E. (1978) Research diagnostic criteria: rationale and reliability. *Arch Gen Psychiatry.* **35**(6):773-82.
7. Manchia M, Adli M, Akula N, Ardau R, Aubry JM, Backlund L, Banzato CE, Baune BT, Bellivier F, Bengesser S, Biernacka JM, Brichant-Petitjean C, Bui E, Calkin CV, Cheng AT, Chillotti C, Cichon S, Clark S, Czerski PM, Dantas C, Zompo MD, Depaulo JR, Detera-Wadleigh SD, Etain B, Falkai P, Frisén L, Frye MA, Fullerton J, Gard S, Garnham J, Goes FS, Grof P, Gruber O, Hashimoto R, Hauser J, Heilbronner U, Hoban R, Hou L, Jamain S, Kahn JP, Kassem L, Kato T, Kelsoe JR, Kittel-Schneider S, Kliwicki S, Kuo PH, Kusumi I, Laje G, Lavebratt C, Leboyer M, Leckband SG, López Jaramillo CA, Maj M, Malafosse A, Martinsson L, Masui T, Mitchell PB, Mondimore F, Monteleone P, Nallet A, Neuner M, Novák T, O'Donovan C, Osby U, Ozaki N, Perlis RH, Pfennig A, Potash JB, Reich-Erkelenz D, Reif A, Reininghaus E, Richardson S, Rouleau GA, Rybakowski JK, Schalling M, Schofield PR, Schubert OK, Schweizer B, Seemüller F, Grigoroiu-Serbanescu M, Severino G, Seymour LR, Slaney C, Smoller JW, Squassina A, Stamm T, Steele J, Stopkova P, Tighe SK, Tortorella A, Turecki G, Wray NR, Wright A, Zandi PP, Zilles D, Bauer M, Rietschel M, McMahon FJ, Schulze TG, Alda M. (2013) Assessment of Response to Lithium Maintenance Treatment in Bipolar Disorder: A Consortium on Lithium Genetics (ConLiGen) Report. *PLoS One.* **8**(6):e65636.
8. Manchia M, Adli M, Akula N, Ardau R, Aubry JM, Backlund L, Banzato CE, Baune BT, Bellivier F, Bengesser S, Biernacka JM, Brichant-Petitjean C, Bui E, Calkin CV, Cheng AT, Chillotti C, Cichon S, Clark S, Czerski PM, Dantas C, Zompo MD, Depaulo JR, Detera-Wadleigh SD, Etain B, Falkai P, Frisen L, Frye MA, Fullerton J, Gard S, Garnham J, Goes FS, Grof P, Gruber O, Hashimoto R, Hauser J, Heilbronner U, Hoban R, Hou L, Jamain S, Kahn JP, Kassem L, Kato T, Kelsoe JR, Kittel-Schneider S, Kliwicki S, Kuo PH, Kusumi I, Laje G, Lavebratt C, Leboyer M, Leckband SG, Lopez Jaramillo CA, Maj M, Malafosse A, Martinsson L, Masui T, Mitchell PB, Mondimore F, Monteleone P, Nallet A, Neuner M, Novak T, O'Donovan C, Osby U, Ozaki N, Perlis RH, Pfennig A, Potash JB, Reich-Erkelenz D, Reif A, Reininghaus E, Richardson S, Rouleau GA, Rybakowski JK, Schalling M, Schofield PR,

Schubert OK, Schweizer B, Seemuller F, Grigoroiu-Serbanescu M, Severino G, Seymour LR, Slaney C, Smoller JW, Squassina A, Stamm T, Steele J, Stopkova P, Tighe SK, Tortorella A, Turecki G, Wray NR, Wright A, Zandi PP, Zilles D, Bauer M, Rietschel M, McMahon FJ, Schulze TG, Alda M. (2013) Assessment of Response to Lithium Maintenance Treatment in Bipolar Disorder: A Consortium on Lithium Genetics (ConLiGen) Report. *PLoS One.* **8**(6):e65636.

9. Boyle AP, Hong EL, Hariharan M, Cheng Y, Schaub MA, Kasowski M, Karczewski KJ, Park J, Hitz BC, Weng S, Cherry JM, Snyder M. (2012) Annotation of functional variation in personal genomes using RegulomeDB. *Genome Res.* **22**(9):1790-7.
10. Dong S, Zhao N, Spragins E, Kagda MS, Li M, Assis P, Jolanki O, Luo Y, Cherry JM, Boyle AP, Hitz BC. (2023) Annotating and prioritizing human non-coding variants with RegulomeDB v.2. *Nat Genet.* **55**(5):724-726.
11. Hodge RD, Bakken TE, Miller JA, Smith KA, Barkan ER, Graybuck LT, Close JL, Long B, Johansen N, Penn O, Yao Z, Eggermont J, Hollt T, Levi BP, Shehata SI, Aevermann B, Beller A, Bertagnolli D, Brouner K, Casper T, Cobbs C, Dalley R, Dee N, Ding SL, Ellenbogen RG, Fong O, Garren E, Goldy J, Gwinn RP, Hirschstein D, Keene CD, Keshk M, Ko AL, Lathia K, Mahfouz A, Maltzer Z, McGraw M, Nguyen TN, Nyhus J, Ojemann JG, Oldre A, Parry S, Reynolds S, Rimorin C, Shapovalova NV, Somasundaram S, Szafer A, Thomsen ER, Tieu M, Quon G, Scheuermann RH, Yuste R, Sunkin SM, Lelieveldt B, Feng D, Ng L, Bernard A, Hawrylycz M, Phillips JW, Tasic B, Zeng H, Jones AR, Koch C, Lein ES. (2019) Conserved cell types with divergent features in human versus mouse cortex. *Nature.* **573**(7772):61-68.
